# Child vaccination in animated infographic: technology for permanent education about the nursing process

**DOI:** 10.1590/1980-220X-REEUSP-2022-0423en

**Published:** 2023-06-09

**Authors:** Fernanda Medrado de Souza Ferreira, Francislene do Carmo Silva, Taison Regis Penariol Natarelli, Débora Falleiros de Mello, Luciana Mara Monti Fonseca

**Affiliations:** 1 Universidade de São Paulo, Escola de Enfermagem de Ribeirão Preto, Departamento de Enfermagem Materno-Infantil e Saúde Pública, Ribeirão Preto, SP, Brazil.

**Keywords:** Immunization Program, Nursing Proces, Educational Technolog, Education, Continuin, Programas de Inmunización, Proceso de Enfermería, Tecnología Educacional, Educación Continua, Programas de Imunização, Processo de Enfermagem, Tecnologia Educacional, Educação Permanente

## Abstract

**Objective:**

To develop and validate an animated infographic about the nursing process in childhood vaccination.

**Method:**

Methodological study for the development and validation of educational technology, an animated infographic, on childhood vaccination. First, contents from the Ministry of Health that should compose the infographic were selected. Then, a script was prepared and a storyboard used to guide the production of the animated infographic. Once finalized, the technology went through the content and appearance validation process with nursing experts in the study area.

**Results:**

Sixty-nine screens of storyboard were done and the infographic lasted five minutes and 52 seconds. Forty-five nurses were selected and, of these, 21 agreed to participate in the study. The infographic was evaluated according to its objectives, structure, presentation, and relevance, resulting in an overall CVI of 97%.

**Conclusion:**

The animated infographic produced was validated by experts and, once adapted following the judges’ suggestions, it became a valid educational tool to be used by students and nursing professionals.

## RESUMO

**Objetivo:**

Desenvolver e validar um infográfico animado sobre o processo de enfermagem na vacinação infantil.

**Método:**

Estudo metodológico para desenvolvimento e validação de uma tecnologia educacional, do tipo infográfico animado, sobre vacinação infantil. Primeiramente, foram selecionados conteúdos do Ministério da Saúde que deveriam compor o infográfico. Em seguida, foi construído um roteiro e utilizado um *storyboard* para nortear a produção do infográfico animado. Após finalizada, a tecnologia passou pelo processo de validação de conteúdo e aparência junto a enfermeiros *experts* na área de estudo.

**Resultados:**

Foram elaboradas 69 telas de *storyboard* e o infográfico apresentou duração de cinco minutos e 52 segundos. Foram selecionados 45 enfermeiros e destes, 21 aceitaram participar do estudo. O infográfico foi avaliado segundo os objetivos, estrutura, apresentação e relevância, resultando em um IVC global de 97%.

**Conclusão:**

O infográfico animado produzido foi validado pelos *experts* e, ao adequar-se às sugestões dos juízes, tornou-se uma ferramenta educativa válida a ser utilizada por estudantes e profissionais de enfermagem.

## RESUMEN

**Objetivo:**

Desarrollar y validar una infografía animada sobre el proceso de enfermería en la vacunación infantil.

**Método:**

Estudio metodológico para el desarrollo y validación de una tecnología educativa, del tipo infografía animada, sobre vacunación infantil. En primer lugar, se seleccionaron los contenidos del Ministerio de Salud que debían componer la infografía. Luego, se construyó un guion y se utilizó un *storyboard* para guiar la producción de la infografía animada. Una vez finalizada, la tecnología pasó por el proceso de validación de contenido y apariencia con enfermeros expertos en el área de estudio.

**Resultados:**

Sesenta y nueve pantallas de *storyboard* fueron elaboradas y la infografía duró cinco minutos y 52 segundos. Fueron seleccionados 45 enfermeros y de estos, 21 aceptaron participar del estudio. La infografía fue evaluada de acuerdo a sus objetivos, estructura, presentación y relevancia, resultando en un IVC global del 97%.

**Conclusión:**

La infografía animada producida fue validada por expertos y, al adaptarse a las sugerencias de los jueces, se convirtió en una herramienta educativa válida para ser utilizada por estudiantes y profesionales de enfermería.

## INTRODUCTION

Vaccination is an action linked to Primary Health Care (PHC) as a preventive care, for health promotion and protection, being an important evaluation indicator that has given great contribution to the control of vaccine-preventable diseases and to the reduction of child mortality^(1)^. PHC is the most strategic level of care for the prevention of diseases and injuries, one of its main characteristics being the first contact access for users of the Brazilian Public Health System (*SUS*). The bond between the user and the health units becomes effective in preventive actions and facilitates the user’s approach to health services^(2)^.

Despite all existing vaccination strategies, vaccination coverage has to be addressed, as some diseases in the elimination or control phase have periods of high incidence. According to the World Health Organization (WHO), the number of measles cases increased by 300% worldwide in the first months of 2019 compared to the same period in 2018^(3)^. The Pan American Health Organization (PAHO) has placed Brazil at high risk of reintroducing polio in the country due to the sharp drop in vaccination, the lowest since 1994. In addition, New York declared emergency in an attempt to accelerate efforts to vaccinate residents against polio after the virus was detected in sewage samples^(4)^.

According to the Ministry of Health, false news, called *fake news*, continue to be used to manipulate, deceive, and harm the population, having been strengthened with the increased use of social media and messaging applications in recent years^(5)^. Although being an old phenomenon, disinformation has reached large proportion due to the emergence of social media and, in particular, the ease and speed of dissemination. In addition to the technological revolution, the uncertainties of the population regarding traditional institutions, among them the media, politics, justice, and the State, are seen as a factor that stimulates the worsening of the problem. The rumors disseminated on the internet lead to lack of interest and unfounded concerns regarding several subjects, among them, vaccination^(6)^.

Health workers’ knowledge is one of the most critical points related to vaccine acceptance by parents; therefore, continuing education regarding routine immunization, adverse events, and the constant search for safety and quality of care provided is extremely important. Failures in immunization occur due to lack of professionals’ skills, related to lack of knowledge and poor qualification. This issue results in incorrect guidance to patients and favors the spread of myths and beliefs, as well as loss of continuity in the vaccination schedule and problems such as delays and loss of vaccine^(7)^. Health unit professionals shall be prepared to work as vaccination educators, as they are seen as a reliable source of information. On the other hand, inadequate or vague information can compromise parental confidence and lead to vaccine hesitancy^(8)^.

The Nursing Process (NP) contributes to the organization of nursing care in the context of health care in any place where professional practice takes place and allow nurses to provide individualized care. Centered on Basic Human Needs, the NP also assists in decision-making in various situations experienced by nurses as managers of the nursing team^(9)^.

Educational Technologies in Health (*TES*) are considered tools that facilitate the teaching-learning process and that contribute greatly to improvements in the quality of care^(10)^. Technologies, when used correctly, can be benefic to the practice of caring for human beings in several ways. Nursing has been committed to the production and search for technological products that can help in their daily professional life, be they care, administrative or educational activities^(11)^.

In their turn, infographics can be defined as graphic visual representations that aim to convey data, knowledge or information quickly and clearly. This way, information considered complex can be more easily communicated to the target audience, through various platforms, such as websites, social media, and television. Animated infographics have already proven to be effective technological resources, capable of facilitating the understanding and reflection of several health topics^(12)^. The care with the set of images, the language used, the sounds and script make the material creative and original, but also convey the idea of responsibility and social commitment^(13)^.

Currently, the use of technologies in people’s daily lives, including the work environment, is undeniable; therefore, we shall take advantage of this technological advance to make a positive contribution and convey knowledge to people. In this regard, the development of an animated infographic on immunization of children can provide answers to the current needs of a new profile of academics and health professionals who seek knowledge through technology. Thus, the present study aimed to develop and validate the content and appearance of an animated infographic, with a technical focus on the nursing process in childhood vaccination.

## METHODS

### Design of Study

This is a methodological study of technological production and validation. This study proposes the construction and validation of the content and appearance of a digital educational material, an animated infographic, with a technical approach, to streamline the learning of students and nursing professionals and enhance the NP in vaccination. This way, the study was developed in two stages: selection of contents to be introduced in the proposed material and development of the animated infographic on childhood vaccination, and validation of content and appearance of the technology produced.

### Stage 1 – Development of the Animated Infographic

For the development of this Virtual Learning Object (VLO), the following steps were followed: planning (analysis and diagnosis and instructional planning) and production (didactic design, media production, review and validation)^(14)^.

To select the content and start building the script, bibliographical readings were performed and websites and manuals from the Ministry of Health were accessed to find important points and appropriate content to be introduced in the infographic. The production of knowledge in the area and the researcher’s practical experience in the area of primary health care were considered, which guided the search for information and content for the script preparation. A systematic review of the literature was not carried out, but searches were for support material in the databases with scientific evidence, since the objective of the study was to construct the infographic and not to perform a review method. The information used in the animated infographic was taken from websites of the Ministry of Health, in the *fake news* and immunizations, as well as from the Childhood Vaccination Schedule (0–5 years) and from the epidemiological surveillance manual for post-vaccination adverse events^(5,15,16)^.

Initially, a script was produced based on the briefing sent by the researcher with the subjects that, according to searches in the literature, should be addressed in the animated infographic. The development of the script was thought to follow the stages of the nursing process in child vaccination, which are: data collection, nursing diagnosis, care planning, implementation of actions, and evaluation.

### Stage 2 – Technology Validation

Following the finalization of the infographic, its content and appearance were validated, with the participation of nursing professors from partner Higher Education Institutions (HEIs) (Universidade de São Paulo, Universidade Federal de Goiás, Universidade Federal do Triângulo Mineiro, Universidade Federal de São Carlos, Universidade de Brasília and Universidade Federal de Santa Catarina) and nurses working for the municipal government of Ribeirão Preto/SP, who have clinical experience in vaccination, primary care, and/or theoretical-practical teaching of the subject. For the infographic validation, convenience sampling was used, among non- probabilistic sampling, and for the selection of experts, criteria adapted from Fehring^(17)^ were used. The exclusion criteria were: being a specialist who changed the line of research less than three years before and who no longer works with the theme or those who have been away from professional practice/education for more than two years. The professors were selected through the Lattes Platform of the Directory of the National Council for Scientific and Technological Development (*CNPq*) and the nurses were indicated by the researcher according to their expertise in the area of professional activity, with contact being made electronically (e-mail).

### Data Collection

Data collection was carried out in January and February 2022. Nurse validators (NV), considered experts through selection according to Fehring’s criteria^(17)^, received an invitation via email to participate and, after acceptance, the following documents with instructions for their completion were also sent via email: a) version of the animated infographic; b) instrument with Likert scale evaluation of content and appearance, consisting of the items: objectives, structure/presentation, and relevance, through an adapted instrument^(18)^, so that the participants evaluated, based on the statements, the educational material as totally adequate, adequate, partially adequate, or inadequate.

The script validation instrument was divided into three stages. The first refers to the objectives of the infographic, in which the evaluator observed whether the purposes, goals or the like that one wishes to meet with the use of technology were achieved. The second stage evaluated the structure and presentation of the technology, and the last stage concerns the relevance of the material produced. For each question of the validation instrument, the NV had options 1, 2, 3 and 4 to mark, with option 1- Totally Adequate (TA), 2- Adequate (A), 3- Partially Adequate (PA) and 4 - Inadequate (I). At the end of the instrument, there was still an optional open field for comments and/or suggestions. The NV’s comments and suggestions, regarding the open field for optional filling in the questionnaire, were recorded in longhand and the statements were identified by the letters NV, followed by the number of the order of participation in the study (Example: NV1), to ensure participants’ anonymity.

### Data Analysis and Treatment

The validation data collected were presented by absolute and relative frequency distribution. To assess the agreement among the experts/judges participating in the evaluation, the Content Validity Index (CVI) was calculated. In the evaluation of the animated infographic of this study, the category not reaching the minimum CVI (CVI ≥ 0.80) after the calculation regarding the answers with scores 1 (totally adequate) and 2 (adequate) had to be reformulated and subjected to a new assessment.

### Ethical Aspects

In compliance with ethical and scientific rigor, the research project was submitted to the Research Ethics Committee of the Escola de Enfermagem de Ribeirão Preto – Universidade de São Paulo, as provided for in the Resolution of the National Health Council (CNS) no. 466/2012 regarding the conduction of research involving human subjects. The project was approved on April 5, 2021, under opinion number 4.629.764. All participants, after being clarified about the research objectives, signed the Free and Informed Consent Form (FICF).

## RESULTS

After reading the literature and accessing the websites and manuals of government agencies such as the WHO and the Ministry of Health on the subject, the content was selected according to the stages of the NP in childhood vaccination, as shown below: Data collection (reception and interview to know about the vaccination status, and caregivers’ knowledge, concerns, and doubts); Nursing Diagnosis (child vaccine hesitancy, uncertainties about the protection of vaccines); Care Planning (complete vaccination at each age – vaccination schedule – health education for caregivers/family); Implementation of Actions (guidelines informed by scientific evidence, explanations in accessible language, search for relevant information); Nursing Assessment (verification of doubts and knowledge about childhood vaccination, continuity of care).

As the work of health professionals is not immune to the phenomenon of anti-vaccination movements, we started the infographic script with information from the Ministry of Health website about the fake news on vaccines achieving and influencing more people, so that correct and reliable guidance is passed on to patients and family members. This fake news are: vaccines cause autism; vaccines have a number of harmful and long-term side effects that are still unknown; vaccination can even be fatal; the combination vaccine against diphtheria, tetanus, and pertussis and the polio vaccine cause child sudden death syndrome; vaccine-preventable diseases are almost eradicated in my country, so there is no reason to vaccinate; giving a child more than one vaccine at the same time can increase the risk of harmful adverse events by overloading their immune system; vaccines contain mercury, which is dangerous.

Subsequently, information on the vaccines administered in each age group, route of application, follow-up visits, and general guidelines regarding the child immunization schedule from birth to five years of age recommended by the Ministry of Health were identified^(16)^.

After finalizing the infographic script, the storyboard and the art of animations were done. Initially, the media team sent a proposal for the characters’ visual identity and the setting and, after adjustments, the characteristics of the story location and characters were thus defined: nurse Jessica, patient Rita (mother), baby/child of Rita, and nursing office. Sixty-nine screens of storyboard were prepared and the infographic lasted 5 minutes and 52 seconds.

Initially, 45 nurses who met the criteria for participating in the validation of this study’s infographic were selected. Of these, only 21 nurses accepted participating and returned the email with the signed FICF and the completed assessment instrument. All experts selected (100%) were female, aged between 30 and 61 years. Time since end of the graduate course ranged from seven to forty years. Regarding academic degrees, fourteen (66%) are specialists, three (14%) are masters, three (14%) are PhDs, and one (4%) has a Postdoctoral course.

Most of the items had positive evaluations by the experts and both the values obtained in the partial CVI and in the global CVI were above the recommended minimum value (CVI ≥ 0.80) for the instrument to be considered validated. No item showed CVI ≤ 0.80, with an overall CVI of 97% being achieved. This way, it was not necessary to reformulate the material and submit it to new validation. Table 1 presents the responses obtained in each item of the validation instrument, according to the domains: objectives; structure and presentation; relevance.

**Table 1.** Answers from the expert judges regarding the validation of the animated infographic – Ribeirão Preto, SP, Brazil, 2022.

Items	Totally Adequate	Adequate	Partially Adequate	Inadequate	CVI
**1- Objectives**					
**1.1 The information/content is consistent with the daily needs of the technology target audience.**	16	5	0	0	1.00
**1.2 The information/content is important for the technology target audience’s life and/or work quality.**	19	2	0	0	1.00
**1.3 Invites and/or instigates changes in behavior and attitude.**	15	5	1	0	0.95
**1.4 May circulate in the scientific environment of the area.**	19	0	2	0	0.90
**1.5 Meets the objectives of institutions that serve/work with the technology target audience.**	17	4	0	0	1.00
**Partial total**	86 (82%)	16 (15.2%)	3 (2.8%)	–	0.97
**2- Structure and Presentation**					
**2.1 The technology is appropriate for the target audience.**	19	2	0	0	1.00
**2.2 Messages are clearly and objectively presented.**	17	4	0	0	1.00
**2.3 The information presented is scientifically correct.**	18	2	1	0	0.95
**2.4 The material is appropriate to the target audience’s socio-cultural level.**	15	5	1	0	0.95
**2.5 There is a logical sequence of the proposed content.**	19	1	1	0	0.95
**2.6 The information has well-structured concordance and spelling.**	20	1	0	0	1.00
**2.7 The writing style corresponds to the level of knowledge of the target audience.**	18	2	1	0	0.95
**Partial total**	126 (85.7%)	17 (11.5%)	4 (2.7%)	–	0.97
**3- Relevance**					
**3.1 The themes show key aspects that must be emphasized.**	20	1	0	0	1.00
**3.2 Technology allows generalization and transfer of learning to different contexts.**	19	2	0	0	1.00
**3.3 The technology proposes the construction of knowledge.**	21	0	0	0	1.00
**3.4 The technology addresses the issues the target audience needs to know.**	18	3	0	0	1.00
**3.5 The technology is suitable for use by any professional as a target audience.**	19	1	1	0	0.95
**Partial total**	97 (92.4%)	7 (6.6%)	1 (0.95%)	–	0.99
**Global total**	309 (86.5%)	40 (11.2%)	8 (2.2%)	–	0.97

All suggestions were evaluated by the researchers and those with greater relevance and possibility of being done at the time were accepted. Even with all the items considered valid, the experts’ suggestions on replacing words, adding information, language and grammatical revision, factors considered essential in the production of educational material were accepted. The changes made were: increase in the size of the written information on the nursing process to facilitate reading and inclusion, in the implementation of care, of the steps to obtain complete and safe immunization. We also accepted the suggestions to change the word “swelling” to “edema”, opting for scientific language, since the focus of the infographic is on health professionals/scholars, although the lay public can also benefit from the technology developed. In addition to other minor modifications, such as standardizing the side of the nurse’s badge and the bun attached to the head to avoid distractions when viewing the infographic, information regarding the BCG vaccine scar was added. According to the informative note No. 10/2019, the Ministry of Health follows the WHO recommendations and does not indicate the revaccination of children who did not develop the BCG vaccine scar, since several studies have shown minimal or non-existent evidence of additional benefit with the vaccine repetition. Therefore, it was concluded that the absence of the vaccine scar after vaccination is not indicative of lack of protection.

After the changes made, the final version of the technology was completed. The animated infographic was called Childhood Vaccination and was available in MP4 format on *YouTube* and shared on social media so that it can be disseminated and used by professors, health professionals, and academics as an auxiliary tool in learning about childhood vaccination and the nursing process on the subject.

**Figure 1.**
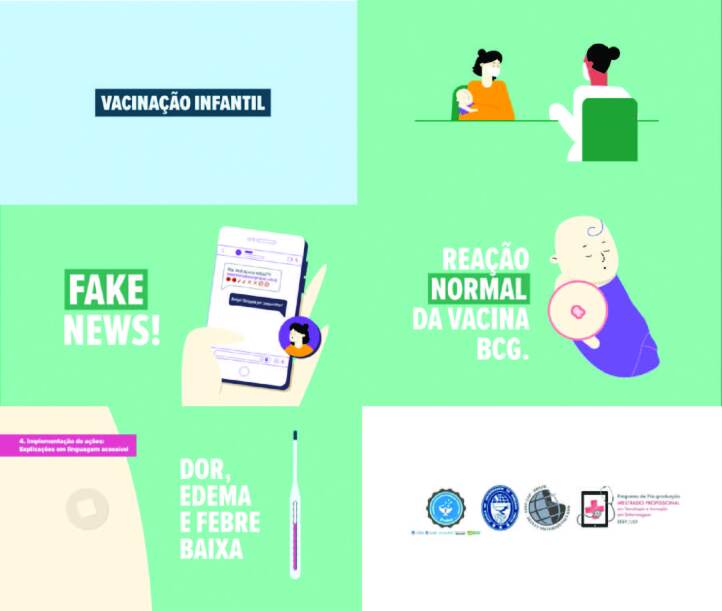
Screens of the animated infographic “Childhood Vaccination”. Ribeirão Preto, SP, Brazil, 2022.

Below, some screens of the finished animated infographic “Children’s Vaccination” will be presented (Figure 1).

## DISCUSSION

The Nursing Process, although being an important strategy for qualifying healthcare/nursing care and services, is still not carried out in several hospitals and in Primary Health Units (*UBS*). Nurses working in PHC perceive a gap between theory and practice, which hinders carrying out the nursing process in their daily work. This way, educational actions, such as Permanent Health Education (*EPS*), are important means to resume the NP in the service to qualify the nursing care provided^(9)^. Furthermore, the authors corroborate the issue in question and emphasize the need to carry out *EPS* specifically on vaccination in PHC. The team’s lack of training and inadequate knowledge about the childhood immunization schedule can result in missed opportunities for vaccination. In a study with PHC health professionals in Montes Claros-MG, lack of training of the teams responsible for vaccination was observed, resulting in difficulties in the domains of clinical indication and contraindication, management of side effects and adverse reactions to immunobiologicals^(19)^.

Educational Technologies (ET) can help in the educational process on vaccination and this topic has already been explored in the literature, with positive findings. A study funded by the CDCs evaluated the use of an ET, a documentary video, for the promotion of vaccination against HPV. The study found a significant increase in participant support (n = 64) to HPV vaccine after viewing and discussing the film^(20)^. Equally positive results were achieved in another study that developed and evaluated a course on vaccine administration in the vastus lateralis muscle of the thigh in children, using the platform *Moodle* and obtaining satisfactory evaluations from the 39 students who participated in the course^(21)^. Such findings reinforce the potential of using ET in teaching about immunization, especially digital ones, both for student training and for the population’s health education.

On the other hand, Brazil is considered one of the countries with the highest production, circulation, and use of false news in the world and, among the various pieces of false information present in the media, conspiracies about vaccination stand out. A study carried out among the 100 links with the greatest reach on social media with the keyword vaccine showed that, among the links found, *fake news* accounted for 13.5% of the total links with the highest engagement^(22)^. In view of this, it is important to keep the team trained to deal with these situations, as well as effective communication between health professionals and the population, since the trust of families in professionals is essential for them to be feel safe concerning the information received about the importance of vaccination.

However, it was not just fake news that have become increasingly present in people’s daily lives. Since the beginning of the 21st century, it has been possible to notice growing computerization of current nursing, with the insertion of digital technologies incrementally prevailing in nursing care and teaching^(23)^. Among the technologies most used to support the nursing process, the following stand out: software, such as CIPE^®^ play^(24)^, the Virtual Learning Environments (VLE), such as the PEnsinar Platform^®(25)^, and Virtual Learning Objects (VLO)^(26)^.

In view of the benefits pointed out with the use of information and communication technologies (ICT) in educational processes, the objective of this study in developing an animated infographic of childhood vaccination followed the idea that this technology is close to the reality experienced in the daily work through animation. Moreover, learners have the possibility of viewing the infographic as many times as necessary, allowing them to reflect on the transmitted information and acquire knowledge according to their need and at their own time for absorption.

Infographics are abundant in visual elements such as colors, drawings, which highlight the information that is intended to be conveyed with it. It is an effective technology for transmitting complex information and provides the receiver with a better understanding of the subject and construction of knowledge through mental connections between visual and verbal elements^(27)^.

When dealing with infographics, it is important to stress that their development is based on fundamental knowledge and specific guidelines. Although infographics are not so commonly used in nursing care and teaching practice, it is believed that in the future this technology may transcend classroom walls and also be used as a final product for learning and patient education, as it is proposed in this research^(28,29)^.

One of the difficulties encountered during the construction of the infographic was adjusting its duration. We managed to finish the infographic with a total duration of 5 minutes and 52 seconds. The ideal duration of an animated infographic should not exceed 5 minutes so that the target audience’s attention is not dispersed^(30)^. Thus, it was not possible, at this time, to accept the judges’ suggestions as for addressing more topics or going deeper into some items, since the infographic would be too long and with an excess of information, which could make it tiring.

The validation by experts has been widely used by researchers in technology development projects. In this study, we chose to validate a previous version of the infographic itself and not of the script before its production. This way, the evaluators are able to have a complete view of the final product with illustrations, sounds and animations. However, the changes made by the video editors are a more complex job that demanded more time.

As for the limitations of this study, we can mention the high cost for the development of the animated infographic, which can be an unfavorable factor for the development of new educational technologies. In addition, one can point out as a methodological limitation of this study the collection of data carried out at a distance, since the experts selected took some time to accept and respond. However, because it was held virtually, it allowed the participation of professionals from different locations, adding content and quality to the animated infographic.

## CONCLUSIONS

The development of this study allowed the process of construction and validation of educational material based on the relationship between the gaps found in the work and the importance of scientific knowledge about childhood vaccination. The methodology used contributed to the development of an attractive and easy-to-understand educational technology, which can encourage researchers to develop other educational technologies, both in this area and in others that can promote knowledge and improve the care provided.

The animated infographic developed in this research is relevant, as it is a new educational technology that can help and facilitate the learning of academics, nurses, nursing assistants/ technicians, physicians, and other professionals involved in the care of children and their families/caregivers. As it is freely accessible and free of charge, we hope that the technology produced will be able to bring information to the scientific community and general population.

We also highlight the importance of carrying out studies that evaluate and monitor the application processes of this infographic to verify the results achieved with its use. In addition, as it is an educational technology, it shall periodically undergo revisions to keep it updated and continuously used and enjoyed.

Therefore, it was concluded that the animated infographic produced was validated by the experts and considered a valid tool to be used for the target population of this study, aiming at informing, expanding knowledge, and promoting reflection on the nursing process in childhood vaccination.
